# Death, inequality, and the pandemic in the nation’s capital

**DOI:** 10.1186/s12889-026-28352-7

**Published:** 2026-07-17

**Authors:** Maria L. Alva, Srujana S. Illa, Jaren Haber

**Affiliations:** 1 Department of Health Management and Policy, Georgetown University, Washington, DC USA; 2 McCourt School of Public Policy, Georgetown University, Washington, DC USA; 3 Quantitative Social Sciences, Dartmouth College, Hanover, NH USA

**Keywords:** Mortality, Loss in Life Expectancy, COVID-19, Demographics, Disease Burden

## Abstract

**Background:**

Abrupt changes in mortality rates and life expectancy provide critical insights into how crises like COVID-19 exacerbate health inequalities across demographic groups. Washington, DC, a city marked by substantial socioeconomic and racial diversity, is a valuable case study to examine the pandemic’s impact. Data on all-cause mortality, cause-specific mortality, and life expectancy highlight how pre-existing disparities have widened across sociodemographic groups relative to pre-pandemic levels.

**Methods:**

We analyzed death certificates from 2015 to 2021 obtained from the DC Department of Health’s Vital Records, alongside annual estimates from the Census Bureau’s American Community Survey. Mortality rate trends were assessed by age, sex, race/ethnicity, and geographic location of residence with comparisons anchored in the 2015–2019 baseline period. Using a life table approach, we estimated years of lost life expectancy (LLE) attributable to COVID-19.

**Results:**

In 2020 and 2021, Washington, DC experienced 1,128 and 629 excess deaths, respectively, compared to the annual average over the preceding five years (158 and 94 per 100,000 population). Of these, 689 and 363 deaths were directly attributed to COVID-19 (97 and 54 per 100,000). Accidental and violent deaths disproportionately increased among specific racial and ethnic groups. Excess mortality rates were higher among men than women by approximately 12 and 5% points in 2020 and 2021, with the majority occurring in residents aged 45 and older. Black and Hispanic residents faced significantly elevated excess death rates—286 and 97 per 100,000, respectively—while Hispanic residents experienced the largest proportional increase in 2020, nearly doubling their pre-pandemic rates. Life expectancy in DC declined by 2.7 years in 2020, exceeding the national decline of 1.8 years. The greatest LLE was observed in Hispanic males, while White females experienced the lowest LLE.

**Conclusions:**

Local-level sociodemographic analyses uncover intersectional health inequalities often obscured in national statistics. The demographic disparities in life expectancy observed in Washington, DC underscore deepening longevity gaps with profound intergenerational consequences beyond the immediate morbidity and mortality of the COVID-19 pandemic.

**Supplementary Information:**

The online version contains supplementary material available at https://doi.org/10.1186/s12889-026-28352-7.

## Background

As of December 2022, the CDC has reported more than 1.1 M deaths from COVID-19 in the US [1]. Herein, we quantify how the pandemic has impacted health outcomes in Washington, DC, a city of approximately 712,816 people with over a thousand documented COVID-19 deaths across the first two years of the pandemic [1, 2]. Because DC is a socioeconomically and racially diverse city, the data describing all-cause mortality, cause-specific mortality, and life expectancy provides an informative case study to understand the extent to which the pandemic has widened pre-existing gaps across sociodemographic groups. Washington, DC, has been consistently listed in the top 10 cities for income inequality and racial segregation in the past five decades, together with New York, Chicago, San Francisco, and Los Angeles [3]. This paper quantifies the mortality burden during COVID-19 for the nation’s capital, a large and economically unequal city.

We primarily track all-cause mortality because monitoring and reporting COVID-19- deaths alone has been shown to be limiting. For example, COVID-19 might not have been listed as a cause of death in death certificates because people who died had not been diagnosed or died of other comorbidities and, therefore, under-reported. COVID-19 might have also impacted deaths by increasing mental health disorders, violence, substance abuse [4], and the risk of otherwise preventable deaths made more likely by reduced use of health care services [5]. On the other hand, reductions in mobility and social distancing mandates might have prevented non-COVID deaths for some groups. For example, flu transmission decreased by 20% [6], potentially saving lives. Given these complexities, tracking all-cause mortality allows us to account for changes possibly induced by policy and behavioral responses while avoiding potential measurement bias from focusing on COVID-19 mortality alone.

We also report changes in the documented causes of death pre-and post-pandemic to understand the indirect consequences of COVID-19 on other cause-specific deaths. The contraction of healthcare consumption at the start of the pandemic [7] and the reductions in mobility under social distancing mandates might have impacted deaths in unforeseeable ways, leading to an increase in deaths that are typically preventable [4]. In other words, we anticipate that COVID-19 might increase reported deaths from other causes, either because COVID-19 was under-reported in surveillance data, the pandemic derailed health services on potentially avoidable deaths, or violence and accidental deaths increased. We also anticipate that these excess deaths might not be equally borne across all groups; therefore, we report the demographic characteristics of those more likely to die from them. Historically, the leading causes of death in Washington, DC, have been heart disease, cancer, stroke, and diabetes [8], and the burden of these illnesses has been unequally distributed across DC’s wards (electoral districts), which are highly stratified along socioeconomic and racial lines.

Finally, we compute Loss in Life Expectancy (LLE). Mortality rates and LLE capture different facets of population health. Mortality rates capture the frequency of death in a given year, expressed as deaths per person-year lived. LLE additionally captures the number of potential years of life lost, offering a more nuanced picture of mortality than crude mortality rates alone. This is especially important when comparing groups (e.g., by sex or race/ethnicity) that may have markedly different age profiles of excess mortality. Declines in life expectancy can also have far-reaching consequences for human capital, productivity, and intergenerational mobility, particularly in light of the well-documented economic and emotional challenges faced by single-parent families [9] and orphans [10].

## Data and methods

As the primary source of information on mortality across sociodemographic groups, we use death certificates for 2015–2021 from the DC Department of Health’s Vital Records [11]. Death certificates represent DC residents who passed away in DC (rather than elsewhere) and contain information on the deceased person’s name, date of birth, date of death, zip code of residence, race/ethnicity, age, sex, underlying causes, and manner of death. Henceforth we use race interchangeably with ethnicity and Non-Hispanic White, Hispanic, and Non-Hispanic Black individuals as together these make up the majority of the DC population.

For contextual demographic information on DC, we use the Census Bureau’s American Community Survey (ACS) annual estimates [12]. To create ward-level demographic strata, we merge the residential zip codes in the DC death certificates with the wards’ longitude and latitude shape files [13] and ward-level demographic data [14].

### Ethics approval and consent to participate

This study was determined exempted by the DC Department of Health’s Vital Records (DC Dept. Hlth IRB #1–00002318) and Georgetown University (STUDY00002853) IRBs. Two IRBS approved the study reaching the same determination: [1] The Center for Policy, Planning and Evaluation DC Dept. Hlth IORG–0001841, who issued the data, approved the research activities in the study and determined that no consent was required given that secondary research on data was conducted and the review was “exempt” and [2] Georgetown University, where the study PI is based.

### Mortality rates

We compute the all-cause mortality rate by demographic strata (i.e., age, race, sex, and ward) by combining death certificate counts with ACS data. As the ACS represents estimates based on annual data collected from a sample of housing units, these data present a margin of error [15]. We report the ACS confidence intervals in Additional File 1, Appendix A but do not account for the lower and upper bounds of the live population in any given year (denominator) in the mortality rate estimates, because deaths (numerator) represent population values rather than estimates as well as rare events (i.e., changes in the denominator estimates will not affect the overall rate). We also look at the changing demographics of all-cause mortality as well as the changing composition of causes of death between 2015 and 2019 (pre-pandemic) and 2020–2021.

### Loss in Life Expectancy (LLE)

We estimate the impact of COVID-19 on Loss in Life Expectancy across demographic groups in Washington, DC by comparing life expectancy with and without COVID-19 exposure. Specifically, we estimate a counterfactual life table that reflects the mortality profile if individuals had not contracted COVID-19, and compare it to an adjusted life table that incorporates increased mortality risk for those with COVID-19. As a baseline, we use the most recently published sex- and race-specific U.S. life Table (2020) to estimate standard mortality rates [16]. To construct adjusted life tables reflecting the presence of COVID-19 in DC, we integrate three data sources: [1] age- and sex-specific all-cause mortality rates from CDC’s National Center for Health Statistics; [2] COVID-19 prevalence by age, sex, and race from DC case data (Additional File 1, Appendix B); and [3] mortality risk stratified by race for individuals with and without COVID-19 (Additional File 1, Appendix C).

To adjust the all-cause mortality rates, we apply a scaling method that differentiates the mortality risk of those with COVID-19 from those without it. Following Pharoah & Hollingworth (1996), we define a scale-up factor θu, to define baseline mortality rates for the COVID-positive subpopulation:

1 $${\theta\:}_{\mathrm{u}}=\frac{\mathrm{r}}{\mathrm{pr}+(1-\mathrm{p})}$$

where 𝑟 is the relative risk of death for individuals with COVID-19, and 𝑝 is the prevalence of COVID-19 in the subpopulation. Conversely, we apply a scale-down factor $\:{\theta\:}_{d}$ (Gray et al., 2010) to adjust mortality among those without COVID-19:

2 $$\:{\theta\:}_{\mathrm{d}}=\frac{1}{\mathrm{pr}+(1-\mathrm{p})}$$

Relative risk *r* is estimated from cause-of-death data (coded as 1 for COVID-19-related deaths, 0 otherwise), while COVID prevalence *p* is derived from confirmed case counts in DC from January 2020 to December 2021 [17].

These scaling factors allow us to simulate a COVID-adjusted life table for DC, from which we can estimate life expectancy conditional on COVID status, and thus compute LLE as the difference in life expectancy between the two scenarios.

Using CDC life tables, we estimate the number of person-years lived between age x and x + n, denoted as n*L*_*x*_ using the standard approximation under the assumption of a uniform distribution of deaths within the interval:

3 $${\;}_{\mathrm{n}}{\mathrm{L}}_{\mathrm{x}}=\mathrm{n}*{\mathrm{l}}_{\mathrm{x}+\mathrm{n}}+\frac{\mathrm{n}}{2}\:{\;}_{\mathrm{n}}{\mathrm{d}}_{\mathrm{x}}$$

Where n is the width of the age interval (e.g., 1 for one-year intervals), $\:{l}_{x}$ is the number of individuals surviving to exact age x, and $\:{d}_{x}$ is the number of deaths between ages x and x + n.

We then calculate the total number of person-years remaining above age x (T_x_) by cumulating the number of person-years lived across all subsequent age intervals, from the oldest to the youngest age group. The average remaining lifetime (life expectancy) at age x is then calculated as: $\:{e}_{x}={T}_{x}/{l}_{x}$, where $\:{l}_{x}$ is the number of individuals surviving to age x. For individuals who die within the age interval x to x+n, Loss in Life Expectancy (LLE) is calculated as the difference between the expected life expectancy in the absence of COVID-19 and the corresponding life expectancy under observed COVID-19 mortality conditions.

## Results

### Mortality rates

**Fig. 1:**
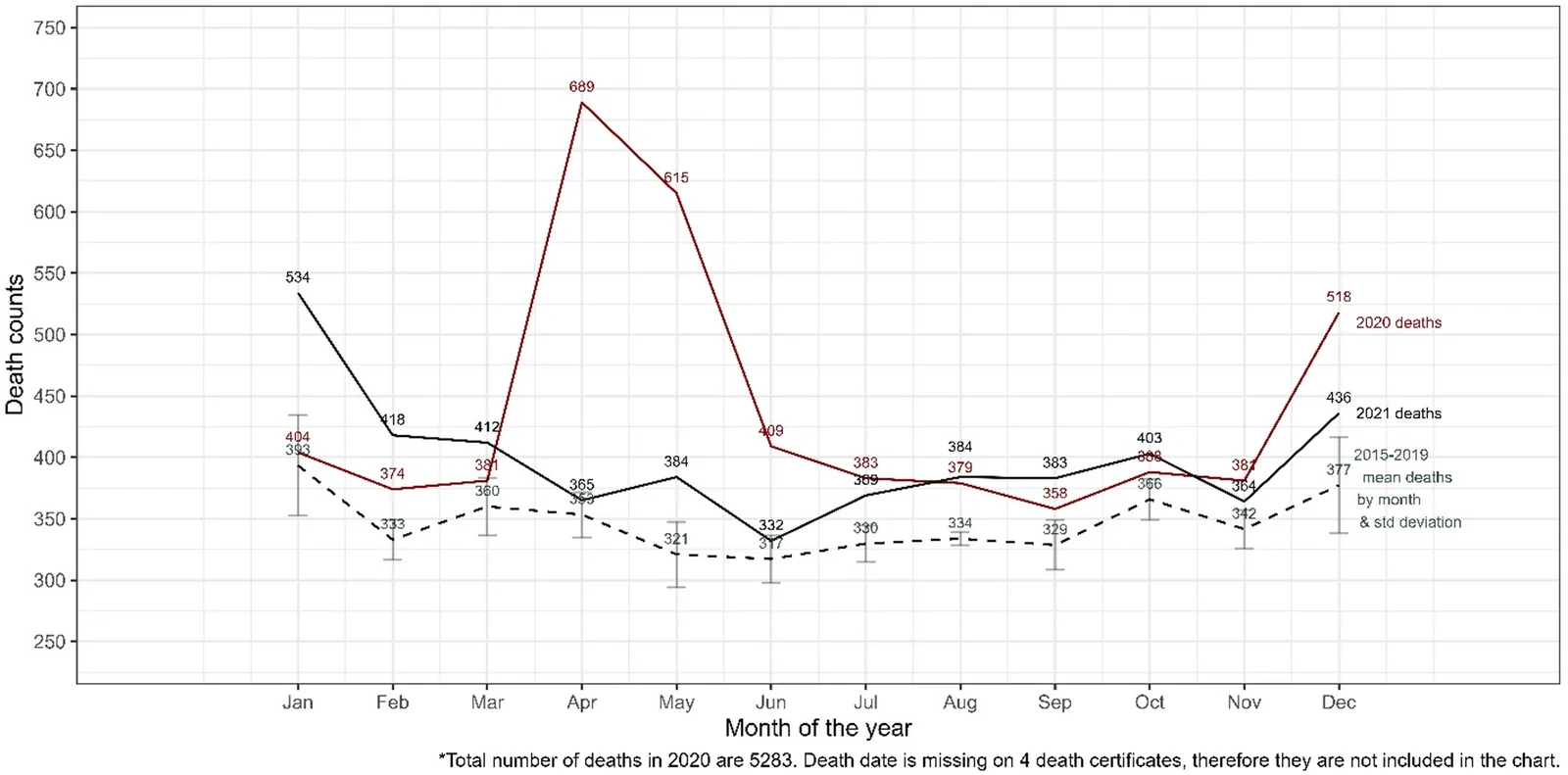
Monthly death counts in Washington, DC, far outpaced previous years in April, May, and December, 2020, and in January, 2021

Figure 1 displays monthly death counts in Washington, DC from January 2020 through December 2021, compared to the historical average from 2015 to 2019. The dotted line represents the five-year monthly average, with whiskers indicating the standard deviation across those years. Notably, in 20 out of the 24 months in 2020 and 2021, death counts exceeded the historical average, often substantially. This excess mortality is most pronounced during the first major COVID-19 wave in April 2020, where deaths peaked at 689, far above the five-year April average of approximately 360. Figure 1 also demonstrates that in the pre-pandemic period (2015–2019), monthly death counts were relatively stable, with minimal variation across years. This stability underscores the significance of the mortality spike during the pandemic years.

In particular, April, May, and December 2020, as well as January 2021, saw spikes of over 50% compared to the same months in previous years. DC recorded its first COVID case on March 7th and its first COVID death on March 20th [18, 19]. The mayor of DC declared the state of emergency on March 11th [20], the same day the World Health Organization announced COVID-19 a worldwide pandemic, and two days before COVID was declared a National Emergency [21]. Schools, indoor venues, and non-essential businesses were closed for the next two months, and large gatherings were banned [22]. Given what we have learned about transmissibility [23] and deaths, it is clear that the first cases happened at least one or two months earlier than officially recorded. Despite the stay-at-home orders, there were 689 deaths in April 2020 and 615 deaths in May 2020—a 95% and 92% increase compared to the 2015–2019 averages for those same months. The simultaneous spread of the Delta and Omicron variants led to the second peak in December 2020, following large-scale infection opportunities including the September 26th White House Rose Garden super-spreader event, Thanksgiving, and end-of-year religious gatherings [24].

Washington, DC, is a city of approximately 700,000 people (Additional File 1, Appendix A) with a well-documented history of economic inequity (Additional File 1, Appendix D). In 2020 and 2021 there were 5,283 and 4,784 deaths, respectively, which translates into 1,128 and 629 excess deaths compared to the previous five years’ average (2015–2019) after accounting for a population changes this translates to 158 and 94 excess deaths per 100 K in 2020 and 2021, respectively. Almost all of these excess deaths were concentrated among residents aged 45 and over. Additional File 1, Appendix E shows the age distribution of the mortality rate per 100,000 people in Washington, DC, from 2015 to 2021.

**Fig. 2:**
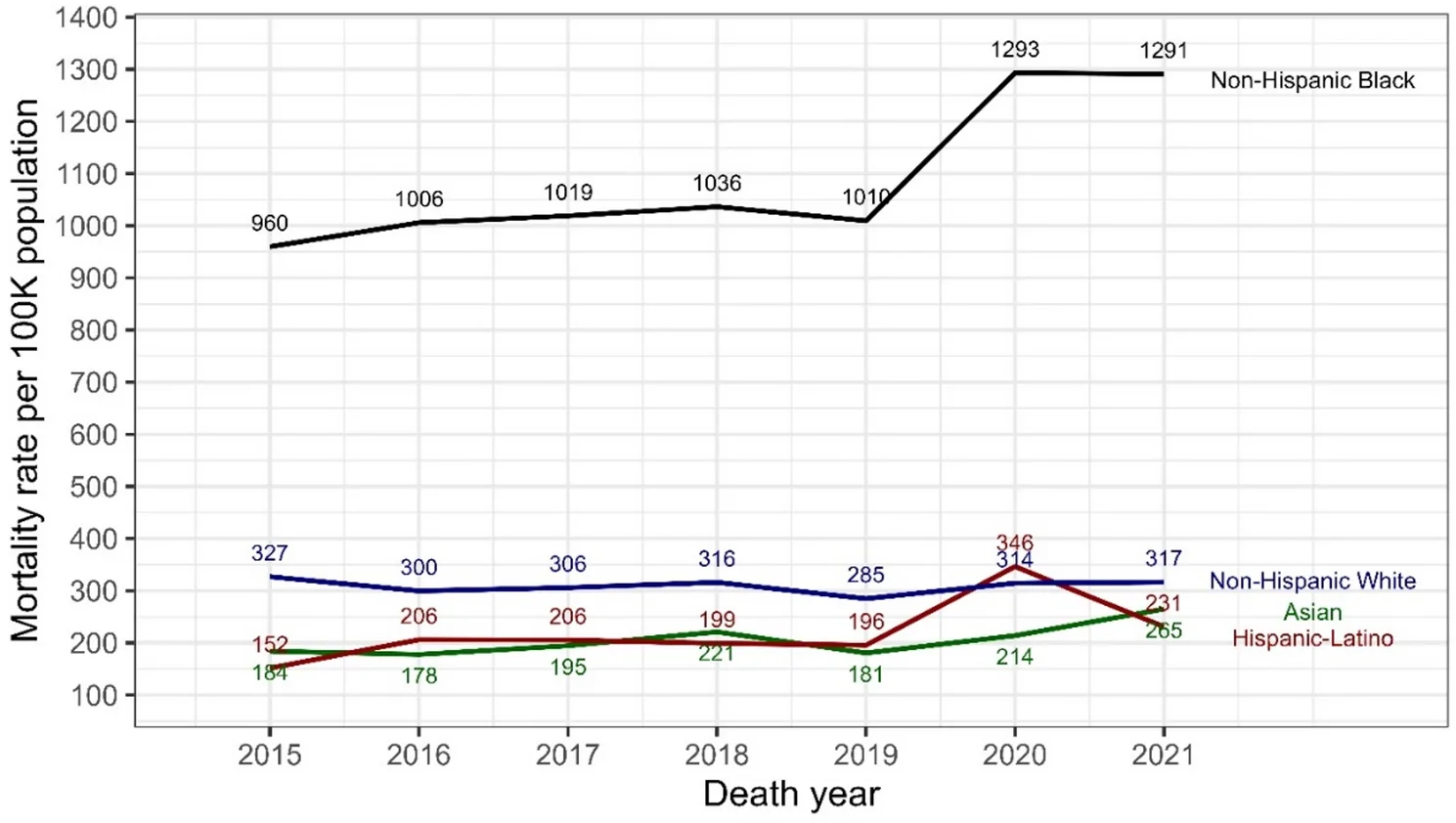
Trends in mortality rates by race and ethnicity in Washington DC. 2015-2021

Figure 2 shows that the already large 2015–2019 mortality gap between the Non-Hispanic Black population and other racial groups increased in 2020 by a further 28% compared to 2019 and sustained at this level in 2021.

Non-Hispanic Blacks represent the majority of deaths across all age groups, with 1,292 deaths per 100,000 in 2020 and 1,291 per 100,000 in 2021, five times the rate of the Non-Hispanic White population. Hispanic/Latinos, on average a socioeconomically disadvantaged group with similar health risks as Blacks, have historically had a longer life expectancy than Black or Whites (a trend called the “Hispanic paradox” [25]). In 2020, however, Hispanics had the highest increase in mortality rates compared to all other groups, an almost twofold increase in mortality compared to the five previous years. In 2021, the Hispanic mortality rate decreased somewhat but did not drop to the pre-pandemic level. For context, DC’s population is 44% Non-Hispanic Black, 37% Non-Hispanic White, 11% Hispanic/Latino, and 4% Asian (see Additional File 1, Appendix A for Census estimates). Over the past ten years, the Non-Hispanic Black population in DC has declined by 5.9%, while the Non-Hispanic White and Hispanic/Latino population has increased by 2.5% and 2.2%, respectively [26].

Inequality in mortality rates has also widened along geographic and socioeconomic dimensions, reflecting racial inequities and residential segregation. For planning and administrative purposes, Washington, DC, is divided into eight wards, each with approximately 88,000 residents. These wards are distinguished from one another by the racial/ethnic groups concentrated therein. The wards with the highest increases in mortality in 2020 relative to the 2015–2019 averages in decreasing order are wards 1 (45%), 7 (28%), 5 (25%), 4 (20%), 6 (18%), 8 (18%), 3 (< 0.5%), 2 (< 0.5%). Wards 2 and 3 are predominantly White, while wards 5, 7, and 8 are mostly Black. Wards 1 and 4 have a relatively large (> 15%) and growing Hispanic/Latino population and are also the most demographically diverse [27]. Wards 4, 5, 7, and 8 are considered low-income communities. Wards 7 and 8, in particular, have unemployment rates over 20%. In contrast, wards 2 and 3 have unemployment rates markedly below the national average [28]. In terms of health outcomes, wards 5, 6, 7, and 8 historically have also had the highest prevalence of chronic conditions in the district [8], and the mortality rate has been highest historically in Wards 5, 7, and 8. While mortality rates decreased in 2021 across all wards, only for wards 1, 2 and 3, rates reverted to pre-pandemic levels (Additional File 1, Appendix F).

**Fig. 3:**
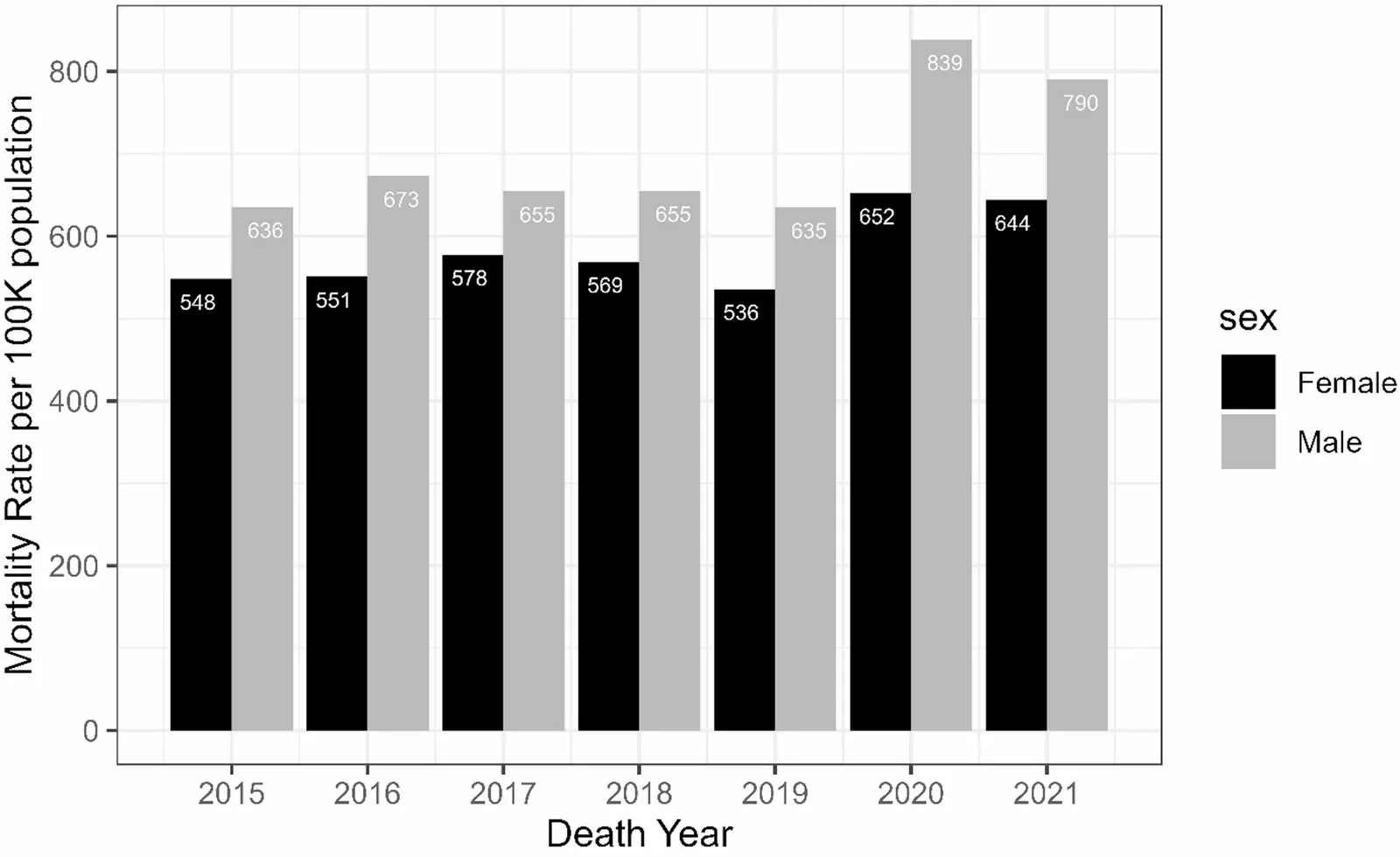
Crude mortality rates in Washington, DC, by sex and year, 2015–2021

Figure 3 shows that the mortality gap by sex also increased in 2020 and 2021. The mortality rate of female residents was 652 deaths per 100,000 in 2020 and 644 per 100,000 in 2021, compared to the male mortality rate of 839 deaths per 100,000 and 790 per 100,000 in 2020 and 2021, respectively.

This means a 17% and 16% increase in mortality for women and a 29% and 21% increase in mortality in mortality for men in 2020 and 2021, respectively, compared to the five-year, pre-pandemic average.

As previously argued, COVID-19 deaths can be under-reported in official statistics because people who died with other comorbidities had not been diagnosed with COVID-19. Our findings support this hypothesis: among DC residents that died in the district in 2020, there were 689 (97 per 100 K) deaths where COVID-19 was listed as a cause of death, and 363 (54 per 100,000) COVID-19 deaths in 2021.

**Fig. 4:**
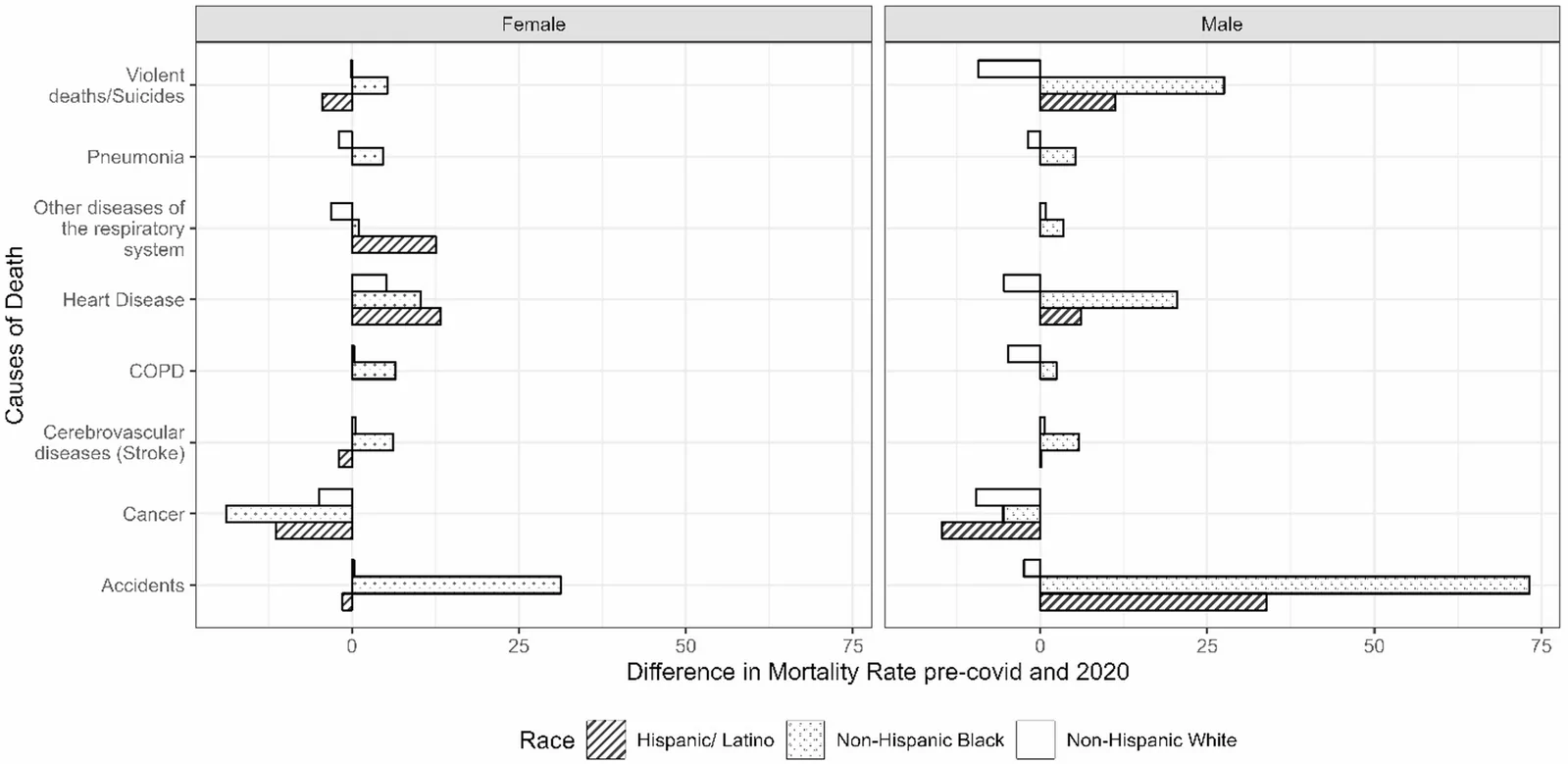
Cause-specific mortality rates grew for Black and Hispanic populations in 2020, particularly among males

Changes in specific causes of death also reveal disparities by gender and race. Figure 4 shows changes in the mortality rate between 2020 and the previous five years combined for some of the most common causes of death in DC by race and sex (Additional File 1, Appendix G shows a very similar pattern for 2021, compared to the 2015–2019 period).

Accidental deaths, violent deaths, suicides, as well as stroke and heart disease-related deaths grew most for Blacks (males and females), and Hispanic males. Communities disproportionately affected by poverty, systemic inequity, and structural racism incurred the heaviest loss of life due to rising gun violence. The Metropolitan Police Department reported a 34% increase in homicides in 2020 compared to the previous five-year average [29] and nationwide, and the CDC reported a 30% increase in the firearm homicide rate since the start of the pandemic [30]. Blacks and Hispanics in the district are also essential and critical infrastructure workers. The U.S. Bureau of Labor Statistics reported increases in numerous construction jobs throughout 2020 [31], which might have contributed to the rise in accidents in groups with higher occupational exposure, like Black and Hispanic males. Some of the increases in mortality related to chronic conditions (e.g., heart disease, stroke COPD, and other respiratory diseases) in 2020 compared to previous years might be due to general avoidance of care linked to the perceived risk of COVID-19 exposure in healthcare facilities [7, 32] as well as the greater vulnerability of Blacks and Hispanics to chronic conditions relative to Whites.

The cause-specific mortality rates for Whites either decreased or stayed the same, irrespective of gender. Decreases in mortality for violent deaths and accidents among White males is likely the byproduct of lockdowns, i.e., the shift from moving out and about in the world to settling into the more protected environment of one’s home. In contrast, decreases in the mortality rate for some of the most prevalent conditions, like cancer and heart disease among the White population, might be the results of a competing risk story. That is, if White people have a relatively higher likelihood of having COVID-19 listed as the primary cause of death, reducing other cause specific death types.

### LLE

The excess deaths entailed a loss of life expectancy for the total population in 2020 and 2021, with larger decreases for Hispanics and Blacks. In 2019, the average life expectancy in Washington, DC, was 78, 0.8 years lower than the national average [33]. The national average life expectancy for Hispanic women was 84.4, for White women was 81.3 years, and for Black women was 78.1 [33]. The national average life expectancy for Hispanic men was 79.1, for White men was 76.3, and for Black men was 71.3. While nationally in the first year of the pandemic we lost on average 1.8 years of life expectancy, DC lost 2.7 years, which again was unevenly distributed (Additional File 1, Appendix H).

Using DC-specific COVID prevalence rates and relative risks of death by race and sex, we estimate that in 2020 White, Black, and Hispanic women experienced, respectively, 1.2, 2.7, and 3.1 LLE due to COVID-19. The estimated LLE for White Black, and Hispanic men in 2020 was 1.5, 3.5, and 4.5, respectively (Table 1).

**Table 1:** Loss in Life Expectancy during the first two years of the pandemic by Race and Sex in DC

Race		Life Expectancy at birth (2020)		Loss in Life Expectancy due to Covid-19 (2020)	Life Expectancy at birth (2021)		Loss in Life Expectancy due to Covid-19 (2021)
	Sex	Without Covid-19	With Covid-19		Without Covid-19	With Covid-19	
Hispanic	Female	84.4	81.3	3.1	81.7	80.5	1.3
Hispanic	Male	79.1	74.6	4.5	75.7	73.9	1.8
Non-Hispanic Black	Female	78.1	75.4	2.7	76.0	74.7	1.2
Non-Hispanic Black	Male	71.3	67.8	3.5	68.4	67.1	1.3
Non-Hispanic White	Female	81.3	80.1	1.2	80.1	79.7	0.4
Non-Hispanic White	Male	76.3	74.8	1.5	74.9	74.4	0.5

While life expectancy improved for all in 2021—possibly due to the arrival of vaccines and the advent of less severe variants—non-Hispanic Black people continued to have higher prevalence rates than the rest of the population, and Hispanics had a much higher risk of death than any other group (Additional File 1, Appendix C). White, Black, and Hispanic women experienced, respectively, 0.4, 1.2, and 1.3 LLE due to COVID-19 in 2021. The estimated LLE for White, Black, and Hispanic men in 2021 was 1.5, 3.5, and 4.5, respectively.

## Discussion

Life expectancy in the United States decreased for the first time in more than two decades during the year 2020 largely due to COVID-19. While there have been fluctuations and temporary decreases in life expectancy in the history of the US due to various factors such as wars, epidemics (i.e., HIV), and economic downturns, the magnitude of the decline experienced during COVID-19 is the biggest since the 1918–1919 Spanish flu pandemic [34]. The widening of the life expectancy gap in a high-income but already unequal city like Washington DC during 2020–2021 emphasizes the importance of micro-level research for grasping the ripples of major health disruptions like the COVID-19 pandemic and supporting population health. Demographic differences in the response to shocks is suggestive of a larger phenomenon where U.S. statistics do not resemble peer nations, especially when we disaggregate data by race, gender, and location. Life expectancy, for example, has not significantly changed (e.g., -0.3 LLE) among OECD countries. In contrast, the U.S. lost, on average, 1.5 life years while DC lost, on average, 2.7 life years. Hispanic males in DC lost 4.5 years, and Black males 3.5 years. The LLE in the Hispanic/Latino and Black population is particularly concerning because it could have severe intergenerational consequences. The premature death of parents and caretakers has been shown, in other contexts, to contribute to social disadvantage across generations due to weathering (the chronic lack of emotional and socioeconomic resources available to children following the death of a parent; [35]), and scarring (reduction in life satisfaction due to an adverse past experience like the death of a parent; [36]).

While we show that national statistics can mask marked differences at the state/city level and within states/cities across groups, in the US, we have not been consistent in reporting sex and race data on COVID-19 prevalence and deaths by location. Only 13 states report cases by sex [37] and only a handful of large metropolitan areas have reported cases by race [38–40]. Comparisons across geography and demographic subgroups depend on access to information on exposure, diagnosis, and demographic information for computing stratified rates. When compared with other states, the overall COVID health profile of DC is below average [41] and when health indicators are stratified by race and wealth proxies (i.e., wards), demographic differences in mortality and LLE are remarkably unequal.

Documenting differences is a first step in determining the causes of gaps. The sex and race gap encountered in the first two years of the pandemic cannot easily explained by any single factor. In the case of sex, biological explanations have been put forward, from the X-chromosome containing a high density of immune-related genes to men being more susceptible to infection due to higher ACE2 plasma levels than women [42]. In the case of race, different health behaviors, from masking to vaccination uptake and occupational risks, ability to self-protect by working from home, prevalence of pre-existing chronic conditions, and access to health care have all contributed in different measures to the existing gaps [43, 44].

Unfortunately, federal funding during the pandemic has not been equitable or commensurate with need. DC has received less funding than Delaware, Rhode Island, Vermont, and Wyoming, which have smaller populations and whose residents pay less in federal taxes [45]. Hospital closures further restrict access to medical resources: Providence hospital, located in Ward 5, was closed in April 2019, and United Medical center, located in Ward 8, is set to close by the end of 2023, with many services halted since 2020, leaving no open hospitals east of the Anacostia River, which only compounds health inequities in Wards 7 and 8. Hispanic Americans remain less likely than other racial and ethnic groups – including White and Black Americans – to be insured or have a primary care provider [46]. Overall, addressing the disproportionate burden of COVID-19 requires a multifaceted approach that addresses the lack of attention and resources currently being devoted to narrow the gap in life expectancy, from better representation from elected officials and community leaders who advocate for their community’s needs to better outreach for vaccinations, prevention of chronic conditions and violent deaths.

## Limitations

Our primary data source underestimates mortality rates in the city. While the denominator represents the count of residents in DC, the numerator represents only DC residents who have died in the district. Local departments of vital statistics do not have access to the death records of residents dying elsewhere. When the CDC aggregates the death counts from states and defines the total counts of deaths for each state and DC, we infer that approximately 14.4% of DC residents have died outside the state or overseas (Additional File 1, Appendix I). Accounting for the demographic characteristics of residents that died outside DC would improve the accuracy of our estimates. Additionally, it is worth stressing that our estimates should be interpreted as differences relative to recent historical patterns, not as model-based projections.

## Conclusion

Our results show that the pandemic is a catalyst of inequality in that its consequences are particularly burdensome on already vulnerable groups. In Washington, DC, the burden of COVID-19 has reversed prior life expectancy gains of Black and Hispanic populations. In the two decades prior to the pandemic, life expectancy increased by 3.9 years, 2.7, and 1.7 years for Blacks, Hispanics and Whites, respectively [47]. Whites have made proportionately fewer gains in life expectancy compared to Black and Hispanics because of the increased mortality among middle-aged white Americans driven by the opioid epidemic [48]. While COVID has played a role in shortening life expectancy, attributing the decline entirely to the virus would be misguided. Data from the Behavioral Risk Factor Surveillance System (BRFSS) has shown an ongoing increase in the prevalence of obesity and chronic conditions among low-income individuals in the district as well as nationally [49]. These trends can create a syndemic context in which infectious and non-communicable disease risks compound one another.

These findings underscore the need for place-based, equity-focused health policies that target both COVID-related risks and the underlying structural conditions—such as unequal access to primary care, housing insecurity, and chronic disease burden—that made certain populations more vulnerable in the first place. Improved data infrastructure at the city and neighborhood level, proactive health outreach, and sustained investment in community health workers and local prevention strategies are crucial for closing these life expectancy gaps.

By devoting resources not only to preventing COVID-specific mortality, but also to addressing the broader social determinants of health, we can reduce long-standing inequities and build a more resilient and equitable public health system.

## Supplementary Information

Supplementary Material 1. Appendix A. Total population estimates in Washington DC by year and racial breakdownAppendix B. COVID-19 Prevalence Rate (%) by Age, Sex, and Race in DC, 2020-2021Appendix C. Relative Risk of COVID Deaths by race in DCAppendix D. Total population estimates by race and wardAppendix E. Between 2020-2021 and the previous five years (2015-2019), excess deaths show that residents 45 and older are most affectedAppendix F. Increases in mortality rate in 2020 were greater for DC wards with more ethnic minorities and lower-income residentsAppendix G. Cause-specific mortality rates by race and sex in 2021, compared to the 2015-2019 periodAppendix H. Differences in Life expectancy in the US between 2019 and 2020: DC lost nearly twice as many years of life expectancy between 2019 and 2020 compared to the US as a wholeAppendix I. DC Department of Vital Statistics versus CDC mortality counts.

## Data Availability

The datasets used and/or analyzed during the current study are available from the corresponding author on reasonable request.

## Abbreviations

LLE: Loss in Life Expectancy
ACS: American Community Survey
BRFSS: Behavioral Risk Factor Surveillance System
