# Supplementary material for: Death, inequality, and the pandemic in the nation’s capital
Source: BMC Public Health. 2026 Jul 17;26:2947. doi: 10.1186/s12889-026-28352-7 (PMC13660219; doi:10.1186/s12889-026-28352-7)
Supplement: Supplementary file 1 [file 12889_2026_28352_MOESM1_ESM.docx]

**Additional File 1**

**Title: Death, Inequality, and the Pandemic in the Nation’s Capital**

**Appendix A. Total population estimates in Washington DC by year and racial breakdown**

| Year | Total population  (% Margin of Error) | % Black  (% Margin of Error) | % White  (% Margin of Error) | % Hispanic  (% Margin of Error) |
| --- | --- | --- | --- | --- |
| 2015 | 672,228 (± 0.1) | 46.5% (± 0.3) | 36.0 % (± 0.1) | 10.6% (± 0.8) |
| 2016 | 681,170 (± 0.1) | 46.4% (± 0.3) | 36.3% (± 0.1) | 10.9% (± 0.7) |
| 2017 | 693,972 (± 0.1) | 45.3% (± 0.4) | 36.5% (± 0.2) | 11.0% (± 0.8) |
| 2018 | 702,455 (± 0.3) | 44.4% (± 0.4) | 36.9% (± 0.1) | 11.3% (± 0.7) |
| 2019 | 705,749 (± 0.1) | 44.1% (± 0.4) | 37.3% (± 0.1) | 11.3% (± 0.7) |
| 2020 | 712,816 (± 0.1) | 44.1% (± 0.4)* | 37.3% (± 0.1)* | 11.3% (± 0.7)* |
| 2021 | 670,050 (± 0.1) | 42.7% (±0.4) | 36.4% (±0.3) | 11.5% (±1.0) |

Source: US Census Bureau (1)

*Note: Because the experimental data tables released by the Census for 2020(2) did not have a breakdown by race, we used the same race composition as 2020.

Margin of Error for Total population and Hispanic population estimates are calculated by aggregating the standard errors of the subgroups.

Standard Error = Margin of Error (90% confidence level)/1.645

Where Margin of Error = max (upper bound - estimate, estimate - lower bound)

$SE\left( \hat{X_{1}}+ \hat{X_{2}}+\ldots\right)$≈ $\sqrt{\left[ SE\left( \hat{X_{1}} \right) \right]^{2}+\left[ SE\left( \hat{X_{2}} \right) \right]^{2}+\ldots}$

Where $SE\left( \hat{X_{1}} \right), SE\left( \hat{X_{2}} \right)$etc. are standard errors of sample estimates and $SE\left( \hat{X_{1}}+ \hat{X_{2}}+\ldots\right)$ is the approximate standard error of the sum of these sample estimates.

## **Appendix B.** **COVID-19 Prevalence Rate (%) by Age, Sex, and Race in DC, 2020-2021**

| Sex | Male | | | Female | | |
| --- | --- | --- | --- | --- | --- | --- |
| Age | Non-Hispanic White | Hispanic | Non-Hispanic Black | Non-Hispanic White | Hispanic | Non-Hispanic Black |
| 0-17 | 4.7% | 16.1% | 23.5% | 4.9% | 16.8% | 24.4% |
| 18-49 | 11.8% | 19.0% | 15.2% | 12.2% | 21.2% | 22.7% |
| 50-65 | 6% | 11.7% | 20.8% | 3.7% | 11.8% | 22.3% |
| 65+ | 5.5% | 6.2% | 17.9% | 3.5% | 5.2% | 17.5% |

Source: CDC case counts (3) and ACS 1-year population estimates (1)

## **Appendix C.** **Relative Risk of COVID Deaths by race in DC**

| Sex | Male | | | Female | | |
| --- | --- | --- | --- | --- | --- | --- |
| Year | Non-Hispanic White | Hispanic | Non-Hispanic Black | Non-Hispanic White | Hispanic | Non-Hispanic Black |
| **2020** | 1.12 | 1.71 | 1.15 | 1.09 | 1.35 | 1.14 |
| **2021** | 1.09 | 1.16 | 1.1 | 1.04 | 1.13 | 1.1 |

Source: Author’s calculations

Relative Risk calculation:

Numerator: number of people dying from COVID-19

Denominator number of people who died from causes other than COVID-19

## **Appendix D.** **Total population estimates by race and ward**

| Ward | Population | % Black | % White | % Hispanic | Median Income |
| --- | --- | --- | --- | --- | --- |
| 1 | 85,289 | 25.4% | 46.9% | 17.6% | $110,457 |
| 2 | 77,923 | 7.9% | 66.1% | 12.9% | $114,649 |
| 3 | 84,651 | 8.1% | 70.4% | 9.6% | $129,681 |
| 4 | 90,300 | 48.4% | 23.6% | 22.5% | $96,245 |
| 5 | 92,094 | 61.3% | 22.5% | 10.4% | $77,367 |
| 6 | 101,882 | 27.7% | 56.3% | 7.8% | $117,752 |
| 7 | 84,271 | 90.2% | 2.7% | 4.0% | $48,474 |
| 8 | 85,564 | 86.0% | 5.9% | 4.1% | $37,963 |

Source: Office of Planning, Open Data DC (4)

Note: Data at the ward level is not available after 2018. Median Income is in 2020 inflation-adjusted dollars.

Note: The three racial groups account for approximately 93% of the population. The remainder represents the following groups as described by the ACS: Asians, American Indian and Alaska Native, Native Hawaiian and Other Pacific Islanders, and Some Other Race.

## **Appendix E:** **Between 2020-2021 and the previous five years (2015-2019), excess deaths show that residents 45 and older are most affected**

## **Appendix F:** **Increases in mortality rate in 2020 were greater for DC wards with more ethnic minorities and lower-income residents**

## **Appendix G:** **Cause-specific mortality rates by race and sex in 2021, compared to the 2015-2019 period**

**Appendix H:** **Differences in Life expectancy in the US between 2019 and 2020: DC lost nearly twice as many years of life expectancy between 2019 and 2020 compared to the US as a whole**

| US | | Life Expectancy at birth | | Years of Life Lost due to Covid-19 |
| --- | --- | --- | --- | --- |
| Race | Sex | Without Covid-19 (2019) | With Covid-19 (2020) |  |
| Hispanic | Female | 84.4 | 81.3 | 3.1 |
| Hispanic | Male | 79.1 | 74.6 | 4.5 |
| Non-Hispanic Black | Female | 78.1 | 75.4 | 2.7 |
| Non-Hispanic Black | Male | 71.3 | 67.8 | 3.5 |
| Non-Hispanic White | Female | 81.3 | 80.1 | 1.2 |
| Non-Hispanic White | Male | 76.3 | 74.8 | 1.6 |
| All National* | | 78.8 | 77.3 | 1.5 |
| DC | | 78.0 | 75.3 | 2.7 |

*Source: National Center for Health Statistics (5)

** Center on Society and Health; National Center for Health Statistics (6, 7)

**Appendix I. DC Department of Vital Statistics versus CDC mortality counts**

| Year | DC Department of Vital Statistics mortality | CDC Mortality (as of 05-01-2023) |
| --- | --- | --- |
| 2015 | 3965 | 4871 |
| 2016 | 4148 | 5037 |
| 2017 | 4262 | 4965 |
| 2018 | 4286 | 5008 |
| 2019 | 4115 | 4927 |
| 2020 | 5283 | 6198 |
| 2021 | 4784 | 5833 |

Sources: DC Department of Vital Statistics (8) and CDC WONDER (9)

Notes: 2020 values are still provisory for the CDC. The CDC numbers include DC residents who die in other states which translated into higher death numbers

**References**

1.	US Census Bureau, ACS 1-Year Estimates Detailed Tables, B02001: RACE. *Census.gov* (November 5, 2022).

2.	US Census Bureau, 2020 ACS 1-Year Experimental Data Tables. *Census.gov* (2021) (August 15, 2022).

3.	CDC, COVID-19 Case Surveillance Public Use Data with Geography | Data | Centers for Disease Control and Prevention (2021) (September 14, 2022).

4.	Office of Planning, Open Data DC, ACS Demographic Characteristics DC Ward. *DC.gov* (August 15, 2022).

5.	E. Arias, J. Xu,, Division of Vital Statistics, United States Life Tables, 2019. 59 (2022).

6.	Center on Society and Health, Washington, D.C. Life Expectancy Methodology and Data Table.

7.	National Center for Health Statistics, District of Columbia (2023) (April 27, 2023).

8.	CDC, NVSS (2022) (August 15, 2022).

9.	CDC WONDER, Mortality Data on CDC WONDER (May 1, 2023).
